# Supplementary material for: Effectiveness of Acute Malnutrition Treatment at Health Center and Community Levels with a Simplified, Combined Protocol in Mali: An Observational Cohort Study
Source: Nutrients. 2022 Nov 21;14(22):4923. doi: 10.3390/nu14224923 (PMC9699530; doi:10.3390/nu14224923)
Supplement: Supplementary file 1 [file nutrients-14-04923-s001.zip › nutrients-1927816-supplementary.pdf]

nutrients

Supplementary Materials

Effectiveness of Acute Malnutrition Treatment at Health Center and Community Levels with a Simplified, Combined Protocol in Mali: An Observational Cohort Study

Suvi T. Kangas [1,*], Bethany Marron [1], Zachary Tausanovitch [1], Elizabeth Radin [1], Josiane Andrianarisoa [2], Salimou Dembele [2], Césaire T. Ouédraogo [2], Issa Niamanto Coulibaly [2], Marie Biotteau [1], Bareye Ouologuem [3], Soumaila Daou [4], Fatoumata Traoré [2], Issiaka Traoré [2], Marc Nene [5] and Jeanette Bailey [1]

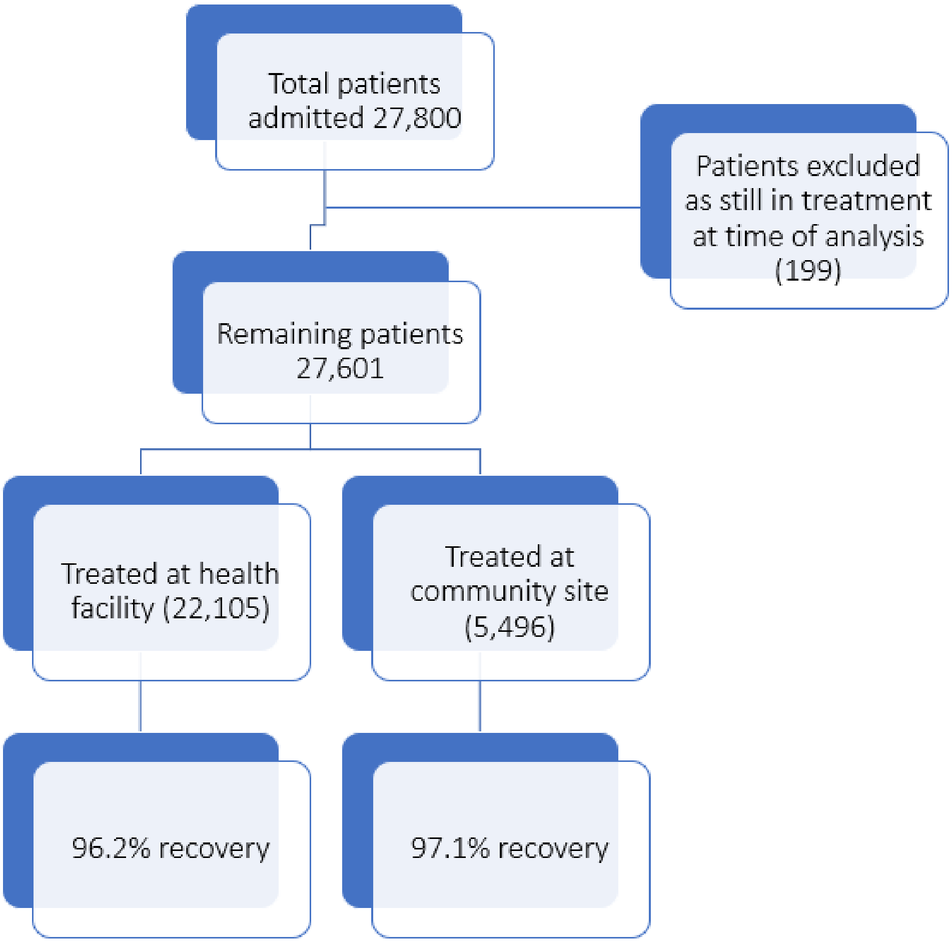

Figure S1. Patient flow chart.

Table S1. Median LOS following simplified treatment by sub-groups.

	Length of stay in days (n) by MUAC & edema category		
	**MUAC <125 mm or edema (All)**	**MUAC <115 mm or edema (SAM)**	**MUAC 115 to <125 mm & no edema (MAM)**
WHZ category			
WHZ < −3	42 (9,629)	56 (5,241)	28 (4,388)
WHZ ≥ −3	28 (12,527)	49 (2,748)	28 (9,779)
Age group			
≥ 24 months	28 (5,817)	49 (1,757)	28 (4,060)
< 24 months	35 (21,784)	56 (7,825)	28 (13,959)
Weight category			
≤ 7 kg	42 (17,642)	56 (8,020)	28 (9,622)
> 7 kg	28 (9,959)	48 (1,562)	28 (8,397)
MUAC category			
< 110 mm	63 (3,472)	63 (3,472)	
≥ 110 mm	35 (24,129)	49 (6,110)	28 (18,019)
WAZ category			
< −3	42 (15,969)	56 (7,680)	28 (8,289)
≥ −3	28 (11,604)	49 (1,874)	28 (9,730)
WaSt			
WHZ < −3 & HAZ < −3	42 (7,958)	56 (4,741)	28 (3,217)
WHZ ≥ −3 & HAZ < −3	35 (4,791)	49 (1,647)	28 (3,144)
WHZ < −3 & HAZ ≥ −3	35 (1,660)	49 (498)	28 (1,162)
WHZ ≥ −3 & HAZ ≥ −3	28 (7,647)	49 (1,072)	28 (6,575)
Screened by			
Health agent at health facility	35 (4,512)	49 (1,793)	28 (2,719)
Community health worker or volunteer	35 (10,478)	56 (3,775)	28 (6,703)
Caregiver (Family MUAC)	35 (11,420)	55 (3,624)	28 (7,796)
Cared at			
Health facility	35 (22,105)	49 (8,046)	28 (14,059)
Community health worker site	35 (5,496)	56 (1,536)	28 (3,960)

Table S2. Characteristics of Defaulted or Non-respondent children.

Characteristic	N not missing	Defaulted	Non-Response
Total, %, (N)	953	845	108
Boys, % (n)	953	43.7% (369)	32.4% (35)
**Characteristics at Admission**			
Age in months, mean [SD]	953	14.5 (7.4)	11.7 (5.8)
Age group, % (n)			
< 24 months	953	81.1% (685)	94.4% (102)
24 months and up	953	18.9% (160)	5.6% (6)
MUAC (mm), mean [SD]	953	111.9 [8.6]	102.4 [11.3]
Weight (kg), mean [SD]	953	6.4 [1.3]	5.3 [1.3]
Height/Length (cm), mean SD	791	70.4 [7.0]	65.6 [6.8]
WHZ, mean [SD]	779	-3.2 [1.4]	-3.5 [1.4]
WAZ, mean [SD]	944	-3.5 [1.3]	-4.3 [1.4]
HAZ, mean [SD]	791	-2.4 [1.9]	-3.2 [1.9]
WHZ category, % (n)			
WHZ <-3	779	53.8% (371)	66.7% (60)
WHZ >-3 & <-2	779	29.2% (201)	21.1% (19)
WHZ >-2	779	17% (117)	10% (9)
Presence of edema, % (n)	953	1.1% (9)	0% (0)
**Post-Treatment Characteristics**			
Referred to inpatient care during treatment, % (n)	953	22.7% (192)	0% (0)
Length of stay (d), mean [SD]	953	36.7 [27.4]	112 [0]
MUAC gain velocity (mm/d), mean [SD]	953	0.2 [0.3]	0.2 [0.1]
Weight gain velocity (g/kg/d), mean [SD]	953	4.1 [7.2]	4.5 [2.8]
Number of RUTF sachets consumed, mean [SD]	953	54.7 [34.9]	171.5 [79.8]
Number of missed visits during treatment, % (n)	953		
None	953	64.9% (548)	26.9% (29)
1 missed visit	953	30.8% (260)	43.5% (47)
More than 1 missed visit	953	4.4% (37)	29.6% (32)

Table S3. T-test of sub-group recovery compared to remaining sample.

Sub-groups	p-values		
	**MUAC <125 mm or edema (All)**	**MUAC <115 mm or edema (SAM)**	**MUAC 115 to <125 mm & no edema (MAM)**
WHZ category			
WHZ < −3	0.000	0.054	0.048
WHZ ≥ −3	0.000	0.054	0.048
Age group			
≥ 24 months	0.004	0.967	0.002
< 24 months	0.004	0.967	0.002
Weight category			
≤ 7 kg	0.000	0.229	0.088
> 7 kg	0.000	0.229	0.088
MUAC category			
< 110 mm	0.000	0.000	
≥ 110 mm	0.000	0.000	
WAZ category			
< −3	0.000	0.015	0.732
≥ −3	0.000	0.015	0.732
WaSt			
WHZ < −3 & HAZ < −3	0.000	0.008	0.516
WHZ ≥ −3 & HAZ < −3	0.070	0.253	0.304
WHZ < −3 & HAZ ≥ −3	0.972	0.080	0.035
WHZ ≥ −3 & HAZ ≥ −3	0.000	0.265	0.202
Screened by			
Health agent at health facility	0.139	0.380	0.979
Community health worker or volunteer	0.017	0.006	0.295
Caregiver (Family MUAC)	0.854	0.189	0.657
Cared at			
Health facility	0.000	0.313	0.021
Community health worker site	0.000	0.313	0.021
